# Entwicklung eines Kompetenzkatalogs für Ärzt:Innen in Weiterbildung zur Curriculumerstellung für die Kreißsaaleinarbeitung

**DOI:** 10.1007/s00101-023-01283-3

**Published:** 2023-05-24

**Authors:** Markus Flentje, Hendrik Eismann, Simon Schwill, Daniel Forstner, Peter Kranke

**Affiliations:** 1 Klinik für Anästhesiologie und Intensivmedizin, Medizinische Hochschule Hannover, Carl-Neuberg-Str. 1, 30625 Hannover, Deutschland; 2 Abteilung Allgemeinmedizin und Versorgungsforschung, Universitätsklinikum Heidelberg, Im Neuenheimer Feld 130.3, 69120 Heidelberg, Deutschland; 3 Lehr‑/AusbZEins, SanLehrRgt Niederbayern, Gäubodenkaserne, 94351 Feldkirchen, Deutschland; 4 Klinik und Poliklinik für Anästhesiologie, Intensivmedizin, Notfallmedizin und Schmerztherapie, Universitätsklinikum Würzburg, Oberdürrbacher Str. 6, 97080 Würzburg, Deutschland

**Keywords:** Anästhesie, Kaiserschnitt, Kreißsaal, Evaluation, Weiterbildung, Anesthesia, Cesarean sectio, Delivery Room, Evaluation, Residency

## Abstract

**Hintergrund:**

Anästhesiologische Expertise ist in der Kreißsaalumgebung an verschiedensten Punkten der Versorgung erforderlich. Die natürliche Mitarbeitendenfluktuation erfordert hierfür eine ständige Fort- und Weiterbildung. In einer ersten Umfrage unter Lehrenden und Lernenden in der Fachärzt:innenausbildung zeigte sich der Wunsch nach einem einsatzortspezifischen Curriculum. Die vorliegende Studie soll im Sinne der spezifischen Lernzieldefinition die notwendigen Kompetenzen für die anästhesiologische Kreißsaalumgebung beschreiben.

**Methodik:**

Mittels einer zweistufigen Online-Delphi-Befragung unter deutschlandweit anästhesiologisch Tätigen im Kreißsaal wurden Zielkompetenzen als Items entwickelt. Diese wurden anschließend in einem größeren Kollektiv auf Relevanz und Validität überprüft und konnten nachfolgend gruppiert werden. In die abschließende Befragung konnten 201 Teilnehmende eingeschlossen werden.

**Ergebnisse:**

In den Priorisierungsprozessen der Delphi-Analyse wurden von den Teilnehmenden nicht priorisierte Kompetenzen, wie z. B. die Neugeborenenversorgung, nicht weiterverfolgt. Nicht alle Faktoren sind darüber hinaus ausschließlich kreißsaalbezogen, wie beispielsweise das Beherrschen des „schwierigen Atemweges“. Nach der Validierung ergab sich ein Kompetenzkatalog mit 8 Skalen mit insgesamt 44 Items (Kayser-Meyer-Olkin-Kriterium 0,88).

**Schlussfolgerungen:**

Es konnte ein Katalog relevanter allgemeiner Lernziele für Ärzt:innen in Weiterbildung entwickelt werden, der allerdings einer Überprüfung auf Vollständigkeit im Kontext der eigenen Arbeitsumgebung bedarf. Kompetenzen, die auch außerhalb der Kreißsaalumgebung erlernt werden könnten, sollten vor einer Kreißsaalrotation erlernt werden. Dies ermöglicht die Konzentration auf bereichsspezifische Items im Rahmen der Kreißsaaleinarbeitung.

## Abstract (en)

**Background:**

Anesthesiologic expertise is used at various points in the delivery room. The natural turnover of professionals requires continuous education and training for patient care. In a first survey among consultants and trainees, the desire for a delivery room-specific anesthesiologic curriculum has emerged. In order to enable a curriculum with decreasing supervision, a competence-oriented catalogue is used in many medical fields. The gain in competence develops gradually. The participation of practitioners should be obligatory to avoid a differentiation between theory and practice. The structural framework of curriculum development by Kern et al. provides the learning objective analysis after further evaluation. In the sense of specific learning objective definition, the present study aims to describe the competences for anesthetists in the delivery room.

**Methods:**

An expert group (active in the anesthesiology delivery room environment) developed a set of items via a two-step online Delphi survey. The experts were recruited from the German Society for Anesthesiology and Intensive Care Medicine (DGAI). We evaluated the resulting parameters for relevance and validity in a larger collective. Lastly, we used factorial analyses to identify factors that could be used to group items into relevant scales. In total, 201 participants took part in the final validation survey.

**Results:**

During the prioritization process of Delphi analyses, competencies such as neonatal care were not followed up. Not all items developed are exclusively delivery room-related, such as managing a difficult airway. Other items are specific to the environment of obstetrics. One example is integration of spinal anesthesia into the obstetric context. Some items are exclusively related to the delivery room, such as in-house standards of care in obstetrics as a basic skill. After validation, a competence catalogue with 8 scales with a total of 44 competence items resulted (Kayser-Meyer-Olkin criterion 0.88).

**Conclusion:**

A catalogue of relevant learning objectives for anesthetists in training could be developed. It specifies the generally required content of anesthesiologic training in Germany. Specific patient groups, such as patients with congenital heart defects, are not mapped. Competencies that could also be learned outside the delivery room, should be learned before the rotation. This enables the focus on the delivery room items, especially for those to be trained who do not work in a hospital with obstetrics. The catalogue needs to be revised for completeness for its own working environment. Particularly in hospitals that do not have a pediatrician available, neonatal care becomes significant. Didactic methods, such as entrustable professional activities, have to be tested and evaluated. These enable competence-based learning with decreasing supervision and reflect the reality in hospitals. As not every clinic can provide the necessary resources for this a nationwide provision of documents would be helpful.

## Hintergrund

Die anästhesiologische Expertise hat in der Arbeitsumgebung „Kreißsaal“ ihre feste Daseinsberechtigung. Der Einsatz im „Kreißsaal“ stellt ein regelmäßiges und herausforderndes Umfeld der anästhesiologischen Tätigkeit in über 600 deutschen Kliniken dar [1]. Die Analgesie unter der Geburt mittels Periduralkatheter (ca. in 22 % aller Geburten in Deutschland) und die anästhesiologische Beteiligung im Rahmen der Durchführung eines Kaiserschnitts (ca. 32 % aller Geburten in Deutschland) sind die häufigsten Gründe der Beteiligung im Behandlungsablauf [2]. Aus anderen westlichen Ländern wird von zunehmenden Komorbiditäten der Schwangeren berichtet [3, 4], sodass die fachlichen Herausforderungen in Zukunft wahrscheinlich steigen werden. Diese Entwicklung spiegelt sich auch in der Publikation von Leitlinien zur Betreuung Schwangerer mit z. B. kardiovaskulären Begleiterkrankungen wieder, die ein Beispiel für komplexe Rahmenbedingungen beschreibt [5]. Die natürliche Fluktuation von Mitarbeitenden im Bereich Anästhesiologie machen eine ständige Aus‑, Weiter- und Fortbildung von Personal für diese Arbeitsumgebung notwendig.

Wesentliche Herausforderungen sind dabei, die Mitarbeitenden anhand der auszuführenden Aufgaben zu befähigen, dass sie am Ende der Einarbeitung ohne weitere Hilfe alle notwendigen Überlegungen und Handlungen durchführen können. Der Erkenntnis folgend, dass diese Entwicklung individuell mit unterschiedlicher Geschwindigkeit verläuft, wurden seit den 1990er-Jahren kompetenzbasierte Ausbildungscurricula für Gesundheitsberufe eingeführt [6, 7]. Diese Umformulierung der Curricula wurde nicht ausschließlich positiv bewertet. Als Kritikpunkt wurde beispielsweise eine Diskrepanz zwischen Kompetenzbeschreibung und den Erfordernissen der täglichen Arbeit benannt [8]. Als Konsequenz daraus sollten Praktizierende aus dem Arbeitsumfeld obligat eingebunden werden.

Das Erreichen einer Handlungskompetenz entwickelt sich in der Regel über verschiedene Niveaus. Ten Cate et al. beschreiben die Kompetenzentwicklung anhand der abnehmenden Intensität für die notwendige Supervision des Ausbildenden [9]. Dieser Idee folgend ist eine Kompetenz nicht ab einem fixen Zeitpunkt vorhanden, sondern es können Stufen in Bezug auf das Kompetenzlevel beschrieben werden. Die Deutsche Gesellschaft für Anästhesiologie und Intensivmedizin (DGAI) definiert in Ihren Empfehlungen drei Kompetenzebenen (Tab. 1).

**Table 1:** 

	Kompetenz Wissen = Kompetenzlevel theoretisches Wissen	Kognitionsdimension	Kompetenz Fertigkeit = Kompetenz in der Praxis (Fertigkeit): (an Modell, Simulator, Schauspielpatient, Patienten)
1	Erkennen und einordnen können (relevante Dinge können)	Erinnern	Assistiert, gesehen haben, demonstriert bekommen
2	Im Alltag damit umgehen (Diagnose, DD, Therapie, etc.)	Verstehen und Analysieren	Anwenden, durchführen können
3	Erweiterte Kenntnisse (u. a. Pathophysiologie, Wirkungsmechanismen)	Evaluieren und Erzeugen	Routine in der Durchführung

*DD* Differenzialdiagnose

**Figure 1:**
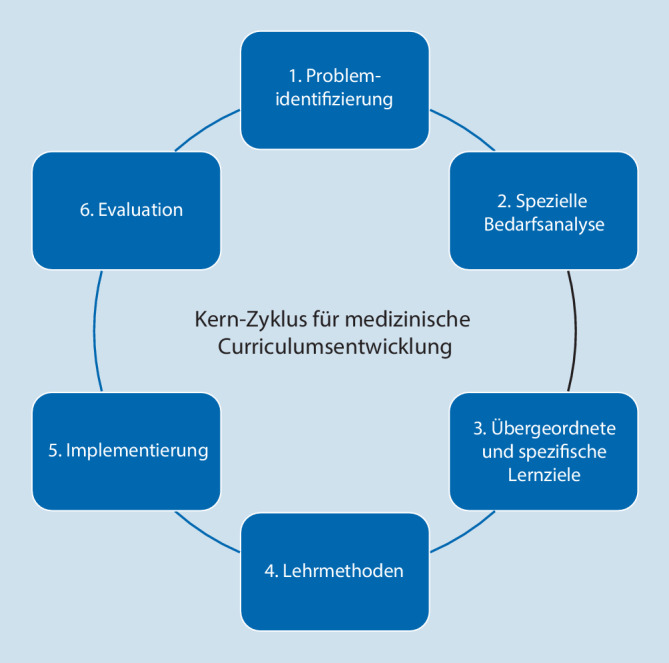

In einer eigenen Umfragestudie konnten die Autoren darstellen, dass Weiterbildende und Ärzt:innen in Weiterbildung (AiW) den Einsatz eines detaillierten Kompetenzkatalogs für die anästhesiologische Kreißsaalumgebung als wünschenswert beurteilen [12]. Die Veränderung des didaktischen Rahmens eines Ausbildungsabschnittes im Rahmen der medizinischen Aus- oder Weiterbildung erfordert nach jedoch einschlägigen Empfehlungen zufolge eine Evaluation und nachfolgend eine kontinuierliche Anpassung [13]. Der in diesem Kontext anerkannte strukturelle Rahmen einer Curriculumsentwicklung von Kern et al. sieht nach der Evaluation die erneute Bedarfs- und Lernzielanalyse vor [13]. Nachdem die Bedarfsanalyse in der zitierten Umfragestudie bereits dargestellt werden konnte [12], legt die vorliegende Studie nunmehr den Fokus auf den Schritt „übergeordnete und spezifische Lernziele“ (Abb. 1).

Ziel der vorliegenden Studie war es, einen detaillierten Kompetenzkatalog für die Tätigkeit von Anästhesiolog:innen in der Kreißsaalumgebung zu entwickeln. Dieser Katalog kann schließlich als Lernzielorientierung und als Basis für weitere didaktische Begleitungen genutzt werden.

## Methodik

### Studiendesign

**Figure 2:**
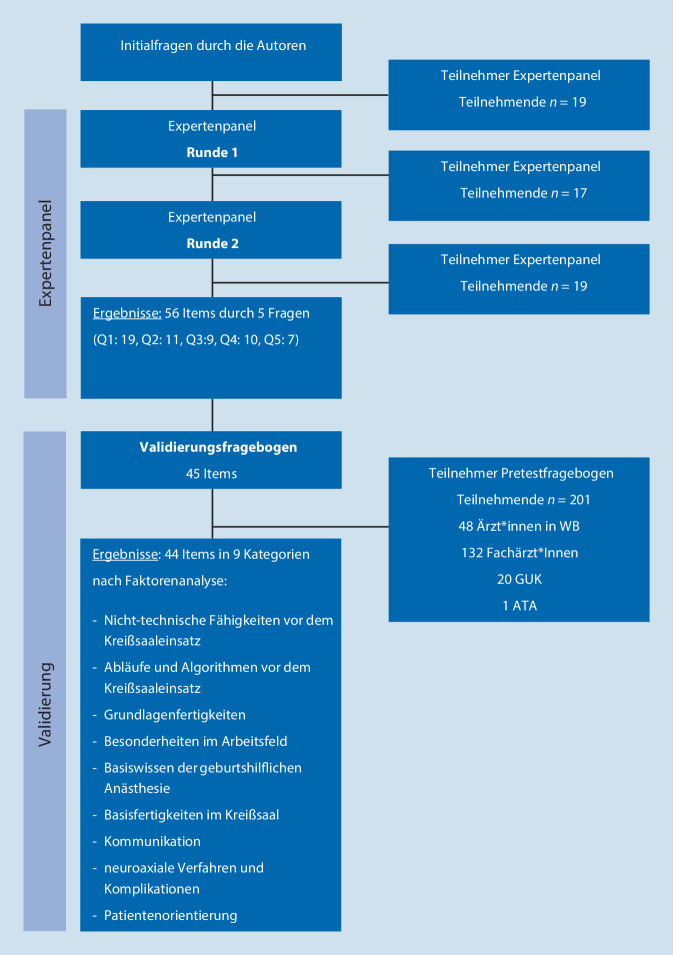

Die Studie wurde als Querschnittstudie geplant. Um die notwendigen Kompetenzen von AiW zu katalogisieren, wurde ein zweistufiges Verfahren eingesetzt, welches bereits in anderen Studien zur Anwendung kam und umfassend beschrieben wurde [14–16]. In Bezug auf die Methoden zum Delphi-Verfahren sind vielfältige Ansätze unter Einbeziehung variabler Stakeholder bzw. Gruppen beschrieben worden [17]. Dieses Vorgehen unterscheidet sich bezüglich Anzahl der Fragerunden, Cut-off-Grenzen und Anwendung der eDelphi-Methode und ist in Abb. 2 näher spezifiziert.

In der ersten Stufe wurden in einer Expertengruppe mit einem elektronischen Delphi-Verfahren notwendige Kompetenzen (eDelphi) für AiW identifiziert und priorisiert. Im zweiten Schritt wurden die identifizierten Items in einer größeren Gruppe mittels Faktorenanalyse validiert und nachfolgend reduziert.

Die Studie wurde von der Ethikkommission der Medizinischen Hochschule und vom Datenschutzbeauftragen genehmigt (9603_BO_K_2021).

### eDelphi-Analyse

Für die erste Runde der eDelphi-Analyse wurden 30 Expert:innen eingeladen. Der Expertenstatus wurde durch die eigene aktive ärztliche Ausbildungstätigkeit oder die Tätigkeit im Funktionsdienst Anästhesie mit fachlicher Zuordnung zum Thema der geburtshilflichen Anästhesie (Pflege/Anästhesietechnische Assistent:in) definiert (jeweils 15 Experten). Die Teilnehmenden wurden nach Begutachtung der Studie durch den wissenschaftlichen Arbeitskreis der DGAI „Geburtshilfliche Anästhesie“ über einen Aufruf der DGAI (Versendung über den E‑Mail-Verteiler) rekrutiert. Zudem wurden die Beteiligten einer Siilo-Gruppe zur geburtshilflichen Anästhesie im DACH-Gebiet (Deutschland, Österreich, Schweiz) nach Interesse im Hinblick auf eine Teilnahme abgefragt.

Der initiale Fragebogen beinhaltete eine Begrüßung, eine Beschreibung des Studienziels, eine Datenschutzerklärung und ein Impressum. Allen Experten wurden die gleichen Initialfragen (Q1–5) mittels Online Tool SoSci (SoSci Survey GmbH, München) gestellt (Tab. 2), die aus der Diskussion im Autorenkreis entstanden sind. Mehrfachnennungen aus vorangehenden Runden wurden von den Autoren zusammengefasst [18]. Eine zweite Runde diente der Priorisierung der benannten Kompetenzen. Den Teilnehmenden wurde zu der jeweiligen Antwort auf Runde 1 alle benannten Antworten in zusammengefasster Version präsentiert. Die Teilnehmenden konnten jeweils bis zu 50 % der möglichen Items für die nächste Runde auswählen.

**Table 2:** 

Fragennummer	Wortlaut der Initialfragen für Delphi-Analyse	Anzahl der Items nach Zusammenfassung
Q1	Welches theoretisch-fachliche Wissen ist aus Ihrer Sicht für einen Assistenzarzt:in der Arbeitsumgebung Kreißsaal wichtig?	34 Items Beispiel: „Vorgehen bei Fruchtwasserembolie“
Q2	Welche technischen Fähigkeiten muss ein eine Assistenzarzt:in der Arbeitsumgebung Kreißsaal vor der Einarbeitung beherrschen?	20 Items Beispiel: „Spinalanästhesie durchführen“
Q3	Welche technischen Fähigkeiten muss ein eine Assistenzarzt:in der Arbeitsumgebung Kreißsaal während der Einarbeitung erlernen?	20 Items Beispiel: „Umgang mit Schmerzpumpen“
Q4	Welche nichttechnischen Fähigkeiten muss ein eine Assistenzarzt:in der Arbeitsumgebung Kreißsaal vor der Einarbeitung beherrschen?	22 Items Beispiel: „Management der Routinesituation“
Q5	Welche nichttechnischen Fähigkeiten muss ein eine Assistenzarzt:in der Arbeitsumgebung Kreißsaal während der Einarbeitung erlernen?	22 Items Beispiel: „Kommunikation im Team mit Geburtshelfer“

### Pretest-Fragebogen

Aus der Priorisierungsrunde wurden insgesamt 118 Items in den Pretest übernommen. Zu allen Items wurde die Frage gestellt, wie wichtig die Kompetenz als Ziel für AiW zur Einarbeitung im Kreißsaal ist. Die Teilnehmenden konnten die Kompetenzen im Hinblick auf Relevanz auf einem Schieberegler bewerten (0 = unwichtig bis 100 = sehr wichtig, Zahlenwerte nicht einsehbar). Die Anzahl der Experten wurde auf 31 Personen aus dem Funktionsdienst Anästhesiologie und 31 anästhesiologisch tätige Ärzt:innen erweitert. Dieses Vorgehen erhöht die Validität und Reliabilität des Tests [19, 20]. Die hinterlegte Skala war dabei für die Teilnehmenden nicht sichtbar. Ziel dieser Bewertungsmethode war es, die Relevanz der Kompetenzen zu bewerten.

### Validierung

Zur Validierung wurde der auf 45 Items gekürzte Kompetenzkatalog eingesetzt. Zur Studienteilnahme wurde mittels DGAI-Mitgliederverteiler aufgerufen und um Einbindung – durch Weiterleitung der Mail – von Teilnehmenden aus „nichtärztlichen“ Berufsgruppen in der jeweiligen Klinik gebeten. Für die Komplettierung der Umfragen hatten die Teilnehmenden jeweils 10 Tage Zeit. Nach 7 Tagen wurde eine Erinnerungsmail verschickt. Die Bewertung der Items erfolgte analog zur Bewertung im Pretest. Nach explorativer Faktorenanalyse wurden die Items aufgrund der Datenlage neuen Faktoren zugeordnet und diese durch die Autoren mit einem übergeordneten Begriff benannt.

### Statistik

Die aufgenommenen Daten wurden nach SPSS 26 (IBM Corporation, Armonk, NY, USA) überführt und dort weiterverarbeitet. In den Priorisierungsrunden wurden die Daten deskriptiv ausgewertet. Zur Beschreibung der Reliabilität der Skalen wurde Cronbachs α berechnet. In der Faktorenanalyse wurde die Eignung der Items mittels Kaiser-Meyer-Olkin(KMO)-Test analysiert. Mittels Kaiser-Gutmann-Kriterium und Varimax-Rotation wurden die Faktoren interpretiert und erstellt [21].

## Ergebnisse

### eDelphi

Die eDelphi-Analyse wurde in 2 Runden durchgeführt. In der ersten Runde nahmen 19 Expert:innen teil (9 Ärzt:innen, 9 Funktionsbereich Anästhesie, 1 ohne Angabe). Nach inhaltlicher Zusammenfassung wurden in Runde zwei 118 Items übernommen. Die Aufteilung nach Initialfragen sind mit Beispielen in Tab. 2 dargestellt.

In Runde 2 nahmen 17 Expert:innen teil. Die notwendige Priorisierung (Auswahl von mindestens 50 % der Expert:innen) erfolgte für die Fragen Q1: *n* = 19; Q2: *n* = 11; Q3: *n* = 9; Q4: *n* = 10 und Q5: *n* = 7 Items.

Beispiele für nichtpriorisierte Items sind „Vorgehen bei Fruchtwasserembolie (Q1)“, „Umgang mit schwierigen PDA/SPA-Verhältnissen (Q2)“, „Säuglingsbasisversorgung (Q3)“, „Kommunikation mit betroffenen Patientinnen (Q4)“ und „klare Kommunikation mit Anästhesiepflege (Q5)“.

### Pretest-Fragebogen

Im Pretest bewerteten 19 der 32 eingeladenen Expert:innen die Items (Abb. 2). Nach Bildung der Mittelwerte und Überprüfung auf Normalverteilung wurde die inhaltsbezogene Überprüfung der Items durchgeführt. Bei ähnlichen Inhalten wurden die Items mit konsistenten bewerteten Mittelwerten und Normalverteilungswerten übernommen. Insgesamt konnten in diesem Prozess 11 Items eliminiert werden; der finale Kompetenzkatalog enthielt 45 Items (Tab. 3).

**Table 3:** 

Faktor	Items gesamt	Items nach inhaltsbezogener Überprüfung	Items nach Reliabilitätsprüfung	Pretest-Fragebogen-Items nach Überprüfung genutzter Antwortkategorie, geringer SD, Trennschärfe
Theoretisch fachlich vor Rotation	19	19	15	15
	α = 0,857	α = 0,857	α = 0,862	α = 0,862
Technisch vor Rotation	11	9	8	8
	α = 0,791	α = 0,764	α = 0,775	α = 0,775
Nichttechnisch vor Rotation	10	8	8	8
	α = 0,904	α = 0,877	α = 0,877	α = 0,877
Technisch während Rotation	9	7	7	7
	α = 0,818	α = 0,801	α = 0,801	α = 0,801
Nichttechnisch während Rotation	7	7	7	7
	α = 0,813	α = 0,813	α = 0,813	α = 0,813

Darstellt sind jeweils die Anzahl der Items und Cronbachs α

*SD* Standardabweichung (standard deviation)

### Validierung

Zur Validierung wurde der auf 45 Items gekürzte Katalog eingesetzt. Die explorative Faktorenanalyse führte zu einer Auswahl von 9 Skalen mit 44 Items, die insgesamt 66,4 % der Gesamtvarianz aufklären konnten (Tab. 4). Das Item „Lernbereitschaft“ wurde aus Faktor 8 zur Erhöhung des Cronbachs α (0,8 statt 0,757) entfernt.

**Table 4:** 

Faktor	Cronbachs α	Anzahl der Items	Anteil der Varianzaufklärung (%)
Nichttechnische Fähigkeiten vor dem Kreißsaaleinsatz	0,865	6	8,61
Abläufe und Algorithmen vor dem Kreißsaaleinsatz	0,776	4	6,47
Grundlagenfertigkeiten	0,744	4	6,45
Besonderheiten im Arbeitsfeld	0,715	4	5,53
Basiswissen der geburtshilflichen Anästhesie	0,918	10	14,48
Basisfertigkeiten für den Kreißsaal	0,872	7	10,42
Kommunikation	0,8	3	5,44
Neuroaxiale Verfahren und Komplikationen	0,757	5	6,03
Patientenorientierung	–	1	2,96
–	–	Gesamtvarianz	66,40
–	–	KMO	0,882

Ein Item wurde im Faktor „Kommunikation“ zur Verbesserung des Cronbachs α entfernt

*KMO* Kaiser-Meyer-Olkin-Wert

Als Prüfgröße für die Eignung des erhobenen Kriterienkatalogs sprach das KMO von 0,882. KMO-Werte > 0,7 werden i. Allg. als gut bewertet [21]. Da die Faktorenanalyse neue Gruppierungen der Items ergab, wurde die initiale Benennung der Faktoren nicht übernommen, sondern die Bezeichnung – nicht zuletzt zur besseren Lesbarkeit und Verwertung im Alltag –von den Autoren neu erstellt (Tab. 5).

**Table 5:** 

Faktor	Items
Nichttechnische Fähigkeiten vor dem Kreißsaaleinsatz	1. Organisationsstrukturen der Arbeitsumgebung kennen 2. Sichere Arbeitsplatzorganisation durchführen 3. Anwendung von Crew-Resource-Management-Inhalten 4. Eine interdisziplinäre Kommunikation mit anderen Fachabteilungen anwenden 5. Im Team (z. B. Hebammen, Geburtshelfer) arbeiten 6. Anlage von i.v.-Zugängen unter erschwerten Bedingungen
Abläufe und Algorithmen vor dem Kreißsaaleinsatz	7. Behandlung hämodynamischer Patienten 8. Behandlung im Kontext Gerinnungsmanagement 9. Narkoseeinleitung mittels Rapid Sequence Induction 10. Algorithmus „Schwieriger Atemweg“ sicher beherrschen
Grundlagenfertigkeiten	11. Sicherer Umgang mit Basismonitoring 12. Sicherer Umgang mit den vor Ort verfügbaren Narkosegeräten 13. Anlage von i.v.-Zugängen unter normalen Bedingungen 14. Eigene Grenzen erkennen/Einschätzen der eigenen Kompetenz
Besonderheiten im Arbeitsfeld	15. Einbinden der Techniken SPA, PDA in den geburtshilflichen Kontext 16. Fachgerechte Anlage von SPA, PDA unter erschwerten Bedingungen 17. Kennenlernen der Alarmierungsketten 18. Zügiges Arbeiten unter Zeitdruck
Basiswissen der geburtshilflichen Anästhesie	19. Physiologische Veränderungen während der Schwangerschaft 20. Medikamentengabe bei Schwangeren 21. Schwangerschaftsassoziierte Pathologien (z. B. Gestosen, HELLP) 22. Ursachen von Blutungen (z. B. Uterusruptur, -atonie, Plazentareste, Geburtsverletzungen, Gerinnungsstörungen) 23. Sectiones caesareae und vergesellschafte Komplikationen 24. Dringlichkeitsstufen bei Sectio caesarea und deren Implikation für die Anästhesie 25. Abwägung von Indikationen SPA, PDA und ITN (Für und Wider in der Geburtshilfe) 26. Anlage von Peridualkathetern und vergesellschaftete Komplikationen 27. Hausinterne Standards in der Geburtshilfe und geburtshilflicher Anästhesie 28. Versorgung des Neugeborenen
Basisfertigkeiten für den Kreißsaal	29. Einschätzen der eigenen Fähigkeiten 30. Kenntnis des eigenen Arbeitsplatzes und daraus resultierende Möglichkeiten sowie Limitationen ableiten 31. Kennenlernen der Standard Operation Procedures im Kreißsaal 32. Rapid Sequence Induction fachgerecht durchführen 33. Schwierigen Atemweg fachgerecht beherrschen 34. Fachgerechte Anlage SPA unter normalen Bedingungen 35. Präzise Kommunikation im Notfall
Kommunikation	36. Im Team mit Geburtshelfer kommunizieren 37. Durchführung der Arbeitsplatzorganisation im Kreißsaal 38. Kommunikation mit Schwangeren
Neuroaxiale Verfahren und Komplikationen	39. Spinalanästhesie fachgerecht durchführen 40. Anlage einer Spinalanästhesie und Beherrschen vergesellschafteter Komplikationen 41. Periduralanästhesie fachgerecht durchführen 42. Anlage einer Periduralanästhesie und Beherrschen vergesellschafteter Komplikationen 43. Reanimation fachgerecht durchführen
Patientenorientierung	44. Empathie mit Patientinnen zeigen

Einige Kompetenzen können auch außerhalb der Kreißsaalumgebung erlangt werden und eignen sich für ein stufenförmiges Curriculum

## Diskussion

In der vorliegenden Studie konnte in einem interprofessionellen Delphi-Prozess ein Kompetenzkatalog für die anästhesiologische Kreißsaalversorgung erfolgreich entwickelt werden, der als Zielgruppe Ärzt:innen in Weiterbildung einschließt.

In der aktuell gültigen WBO Anästhesie werden 3 Items mit Bezug zur Geburtshilfe genannt, die zusammengefasst die Regional- und Allgemeinanästhesie bei Schwangeren, Schmerztherapie in der Geburtshilfe und die Durchführung von 50 Anästhesieverfahren in der Geburtshilfe (25 Kaiserschnitten) fordern [22]. Diese sehr allgemein gehaltenen Anforderungen sind vorteilhaft, wenn der Fokus darauf liegt, möglichst vielen Teilnehmer:innen eine Basisausbildung in einem spezifizierten Schwerpunkt zu ermöglichen. Nachteilig wirkt sich aus Sicht der Autoren die sehr allgemeine Beschreibung auf die im Detail notwendige Ausbildung von Kompetenzen aus, wenn die betreffenden Weiterbildungsteilnehmenden tatsächlich in die geburtshilflich-anästhesiologische Versorgung eingebunden werden sollen. Die von uns in der Vorstudie beschriebene Spannweite der Art der durchgeführten Maßnahmen durch AiW und die positive Bewertung eines potenziellen Curriculums sind hierfür Hinweise [12]. Diese Lücke zwischen Anforderungsprofil einerseits und geforderter Basisausbildung gemäß WBO soll durch die Ergebnisse der vorliegenden Untersuchung und Analyse ansatzweise geschlossen werden.

Aufgrund der initial gestellten Fragen in Bezug zur Zielgruppe der postgraduierten Entwicklung zum Fachärzt:in bildet der entwickelte Katalog neben kreißsaalspezifischen Maßnahmen auch solche Kompetenzen ab, die auch an anderer Stelle erworben werden können. Beispielhaft sei die „Anlage einer Spinalanästhesie unter normalen Bedingungen“ genannt. Bei einer abnehmenden Zahl von Krankenhäusern mit Geburtskliniken [23] ist davon auszugehen, dass die Weiterbildung in steigender Anzahl auch als Rotationseinsatz in Partnerkliniken stattfindet. Daher scheint es legitim bis zwingend erforderlich, dass diese Fähigkeiten bereits an anderer Stelle als dem Kreißsaal erworben werden. Die Erwähnung im Kompetenzkatalog für geburtshilfliche Anästhesie begründet sich dann darauf, dass diese Fähigkeiten vor der Rotation nachgewiesen werden sollten. Dies senkt nach der „cognitive load theory“ die Lernbelastung während der Rotation [24]. Die AiW können sich auf die ausschließlich in der Geburtshilfe zu erlernenden Items fokussieren. Manche Maßnahmen werden in verschiedenen Faktoren benannt (z. B. Periduralanästhesie). Auch diese Zuordnung wurde belassen, um sich der Kompetenz für die Kreißsaalumgebung schrittweise und aus verschiedenen Sichtweisen bzw. Perspektiven zu nähern. Das Erlangen der Kompetenz „Abwägung der Indikation der Narkoseform“ kann beispielsweise für die Einsatzmöglichkeit in der Prämedikation genutzt werden. Dort ist beispielsweise bei Erlangen der Kompetenz keine Supervision auf Sicht notwendig, während die konkrete Anwendung an der Patientin noch einer direkten Supervision bedarf.

Die Basisversorgung des Neugeborenen ist durch den Priorisierungsprozess nicht in den abschließenden Katalog aufgenommen worden. Dies mag erstaunlich wirken, wenn das Behandlungsteam initial ausschließlich aus Anästhesiolog:innen und Geburtshelfer:innen, beispielsweise bei einem Notfallkaiserschnitt, besteht. Der akute Handlungsbedarf erfordert Maßnahmen [25], die das Warten auf pädiatrische Unterstützung nicht erlauben und auch anderweitig in der Ausbildung nicht zwingend erlernt werden. Das im Priorisierungsprozess ausgeschiedene Item „Neugeborenenversorgung“ ist nach unserer Interpretation ein gutes Beispiel für die notwendige kritische Überprüfung und Adaption des Katalogs im Kontext der eigenen Arbeitsumgebung. Ist dort keine initiale neonatologische Versorgung vorhanden, scheint die beschriebene Kompetenz sehr wichtig zu sein, andernfalls nicht. Da wir eine Spezifikation der Klinikstruktur in unserer Umfrage nicht aufgenommen haben, können wir nicht nachvollziehen, ob es zu einer Verzerrung durch Umfrageteilnehmende durch einseitige Strukturen gab. Dieser Interpretation folgend, kann es auch für Fachärzt:innen nach Wechseln des Arbeitsplatzes bedeutsam sein, das eigene Profil nochmals zu überprüfen und ggf. zu erweitern.

Die reine kompetenzorientierte Beschreibung eines Curriculums sieht sich dem Vorwurf ausgesetzt, dass sie zu theoretisch und entfernt von der täglichen Arbeit ist [26]. Diese Lücke kann von ausgewählten didaktischen Methoden, wie beispielsweise der Schaffung von „Entrustable Professional Activities“ (EPA) geschlossen werden [27]. Die didaktische Methode der EPA ermöglicht die schrittweise Übertragung praktischer Fähigkeiten in den klinischen Alltag in Abhängigkeit vom bestehenden Kompetenzlevel. Die Einführung von EPA wird mitunter als wünschenswert erachtet, aber größtenteils noch nicht umgesetzt [28]. Moll-Khosrawi et al. haben im Rahmen einer EPA-Entwicklung für die anästhesiologische Weiterbildung zur Facharzt:innen den Ansatz gewählt, die Weiterbildung nach Möglichkeiten des Einsatzes von EPA zu analysieren. In dieser Untersuchung wurde zwei Situationen aus dem geburtshilflichen Kontext identifiziert (Betreuung einer Sectio: perioperative/peripartale Schmerztherapie) [29]. Einige von uns entwickelte Zielkompetenzen lassen sich in diesen vorgeschlagenen EPA zusammenfassen (z. B. „Einbinden der Technik SPA in den geburtshilflichen Kontext“ und „Empathie mit dem Patienten zeigen“). Hier wäre in einem zukünftigen Praxistest für die entwickelte EPA ein Abgleich zu den von uns beschriebenen Kompetenzen zu empfehlen.

Ein weiteres didaktisches Mittel stellen die sog. Direct Observation of Procedual Skills (DOPS) dar [30]. Diese ermöglichen ein Feedback im Rahmen von arbeitsplatzbasierten Begleitungen nach festen Kriterien und sind z. B. für technische Maßnahmen geeignet. Aus dem Katalog sind hierfür z. B. die Anwendungen wie „Anlage von i.v.-Zugängen unter normalen Bedingungen“ geeignet. Im Arbeitsumfeld der Autoren haben sich bei der Erstellung von DOPS Diskussionen entwickelt, welche Kriterien für eine erfolgreiche Maßnahmendurchführung notwendig sind. Die Autoren sind sich einig, dass die Erstellung eines diesbezüglichen Kataloges für viele Maßnahmen Diskussion im Team auslösen und auf diese Weise einen zusätzlichen interessanten Beitrag für die Weiterbildung leisten wird. Internationale Publikationen zu einzelnen Maßnahmen sind bereits vorhanden und können als Vorlage für die konkrete Umsetzung dienen [10].

Auch die berechtigte Frage nach Konsequenzen aus einer potenziellen Nichterfüllung von Items liegt naturgemäß auf der Hand. Aus Sicht der Autoren bietet der Katalog eine sachliche Information über den Lernfortschritt des Teilnehmenden. Wir sehen ein gutes Potenzial für eine individuelle Lernplanung. Werden Kriterien nicht erfüllt, obliegt es den Weiterbildungsverantwortlichen, über aktuelle Einsatzmöglichkeiten und Erfolg der Weiterbildung zu entscheiden. Tunlichst sollte der Eindruck vermieden werden, es würden durch ein solches Instrument weitere „medikolegale Fallstricke“ in die ohnehin komplexe Thematik zum „Fach:ÄrzInnenstandard“ bzw. zur Delegation medizinischer Leistungen eingezogen werden.

In Bezug auf onlinebasierte Umfragestudien existieren verschiedenste Limitationen, die bei der Interpretation der Aussagen berücksichtigt werden sollten. Es existiert kein den Autoren bekannter Verteiler von anästhesiologischen Kolleg:innen, die einen definierten, hochfrequenten Kontakt zur geburtshilflichen Anästhesie haben. Daher sind eine Rückläuferquote und eine Bewertung der praktischen Erfahrung der Antwortenden im Rahmen der datenschutzrechtlichen Limitationen nicht zu eruieren. Zudem ließen Datenschutzgutachten keine Dokumentation von IP-Adressen zu, was grundsätzlich auch ein Mehrfachausfüllen des Fragebogens ermöglichte. Wir sehen jedoch hierfür bei der Fragestellung keinen Motivationsgrund bei den Teilnehmenden und bewerten das hieraus resultierende Verzerrungsrisiko als gering. Die Rekrutierung der Umfrageteilnehmenden im Umfeld der DGAI kann zu Verzerrungen, insbesondere bei den Priorisierungsprozessen, geführt haben. Als Schlussfolgerung hieraus empfehlen wir den entwickelten Katalog als Basis für weitere Arbeiten und eine Reevaluation nach Erlangung erster Anwendungserfahrungen.

Die anästhesiologische Versorgung im Kreißsaal bedarf einer hohen fachlichen Kompetenz, die schrittweise erlangt werden kann. Mit der Beschreibung von Zielkompetenzen ist es möglich, diese Weiterbildung strukturierter durchzuführen. Die Möglichkeiten, Kompetenzen als Voraussetzungen für Rotationen zu beschreiben, kann zu einem verbesserten Lernerfolg im Kreißsaaleinsatz führen. Die Kompetenzen benötigen im nächsten Schritt didaktische Umsetzungsvorschläge. Die Einführung von EPA kann für bestimmte Teilaspekte eine mögliche Lösung im Rahmen der klinischen Anwendung sein. Da davon auszugehen ist, dass nicht jede Klinik die Ressourcen für deren Entwicklung aufwenden kann, wäre die Bereitstellung landesweiter Lehrunterlagen als Lösungsansatz denkbar. Diese könnten gemäß den konkret vor Ort bestehenden Anforderungen adaptiert werden. Die Anwendung solcher didaktischen Hilfen und Kataloge bedarf im Sinne des Kern-Zyklus nachfolgender Evaluationen.

Abzugrenzen ist das Ergebnis von derzeit noch spezielleren Patientengruppen in der Kreißsaalumgebung, die eine noch weiter gefasste Expertengruppe benötigen würde. Stellvertretend hierfür sei die Versorgung kardial vorerkrankter Patientinnen genannt [31]. Die Relevanz einer solchen weiterführenden Spezialisierung wird seitens der Autoren aufgrund der Kliniklandschaft in Deutschland für wenig zielführend erachtet.

## Fazit für die Praxis

- Der entwickelte Kompetenzkatalog bedarf kritischer Überprüfung, z. B. im Hinblick auf die „Passgenauigkeit“ für die eigene Arbeitsumgebung (u. a. Neugeborenenversorgung).
- Beschriebene Kompetenzen, die nicht zwingend in der Kreißsaalumgebung erlernt werden müssen, sollten vor der Rotation erlernt werden, um den Lerneffekt im eigentlichen Kreißsaaleinsatz zu steigern und den mitunter knapp bemessenen zeitlichen Rahmen bestmöglich zu nutzen.
- Entrustable Professional Activities (EPA) können als didaktisches Mittel zur Entwicklung der Kompetenzen beitragen.
- In einer EPA können mehrere Kompetenzen zusammengefasst werden; welche Kombinationen hier bei sinnvoll sind, muss in der Praxis überprüft werden.
- Die aufgrund der Untersuchungsergebnisse vorgeschlagenen Anregungen dienen als Stütze und Entscheidungshilfe für den transparenten Umgang mit dem erforderlichen Wissen sowie den Kompetenzen und Fertigkeiten für einen spezifischen Arbeitsbereich.
